# Supplementary material for: Effectiveness and Safety of Ayurvedic Medicines in Type 2 Diabetes Mellitus Management: A Systematic Review and Meta-Analysis
Source: Front Pharmacol. 2022 Jun 8;13:821810. doi: 10.3389/fphar.2022.821810 (PMC9213670; doi:10.3389/fphar.2022.821810)
Supplement: Supplementary file 1 [file DataSheet1.zip › Supplementary Material/Forest and Funnel Plots/Sesamum indicum/Fasting insulin.pdf]

Sesamum indicum - Fasting insulin

Study	Intervention N	Intervention Mean	Intervention SD	Comparator N	Comparator Mean	Comparator SD	Mean Diff. with 95% CI	Weight (%)
Shahi 2017	22	36.78	18.57	22	37.28	18.61	-0.50 [-11.49, 10.49]	43.92
Aslam 2018	23	23.13	5.52	23	7.93	1.82	15.20 [12.83, 17.57]	56.08
**Overall**							8.30 [-6.97, 23.58]	

Heterogeneity: $\tau^2 = 106.80$, $I^2 = 86.66\%$, $H^2 = 7.50$

Test of $\theta_i = \theta_j$: Q(1) = 7.50, p = 0.01

Test of $\theta = 0$: z = 1.07, p = 0.29

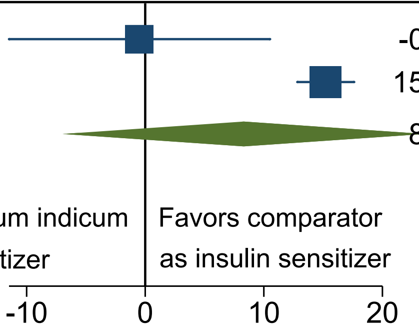

Random-effects REML model
